# Infectious Mononucleosis Triggers Generation of IgG Auto-Antibodies against Native Myelin Oligodendrocyte Glycoprotein

**DOI:** 10.3390/v8020051

**Published:** 2016-02-12

**Authors:** Kristina Kakalacheva, Stephan Regenass, Silke Wiesmayr, Tarik Azzi, Christoph Berger, Russell C. Dale, Fabienne Brilot, Christian Münz, Kevin Rostasy, David Nadal, Jan D. Lünemann

**Affiliations:** 1 Institute of Experimental Immunology, Laboratory of Neuroinflammation, University of Zürich, 8057 Zürich, Switzerland; k.kakalacheva@gmail.com; 2 Department of Clinical Immunology, University Hospital Zürich, 8091 Zürich, Switzerland; s.regenass@balcab.ch; 3 Department of Pediatrics, Paracelsus Medical University Salzburg, 5020 Salzburg, Austria; s.wiesmayr@salk.at; 4 Experimental Infectious Diseases and Cancer Research, University Children’s Hospital of Zürich, University of Zürich, Zürich, Switzerland; Children’s Research Center, University Children’s Hospital Zürich, University of Zürich, 8008 Zürich, Switzerland; azzi.tarik@gmail.com (T.A.); Christoph.Berger@kispi.uzh.ch (C.B.); David.Nadal@kispi.uzh.ch (D.N.); 5 Neuroimmunology Group, Institute for Neuroscience and Muscle Research, The Kids Research Institute at the Children’s Hospital at Westmead, University of Sydney, Westmead NSW 2145, Australia; dr.russell.dale@gmail.com (R.C.D.); fabienne.brilot@sydney.edu.au (F.B.); 6 Institute of Experimental Immunology, Laboratory of Viral Immunobiology, University of Zürich, 8057 Zürich, Switzerland; muenzc@immunology.uzh.ch; 7 Division of Pediatric Neurology, Children’s Hospital Datteln, University Witten/Herdecke, 45711 Datteln, Germany; K.Rostasy@kinderklinik-datteln.de; 8 Department of Neurology, University Hospital Basel, 4031 Basel, Switzerland

**Keywords:** Epstein Barr virus, infectious mononucleosis, autoimmunity, autoantibody, multiple sclerosis

## Abstract

A history of infectious mononucleosis (IM), symptomatic primary infection with the Epstein Barr virus, is associated with the development of autoimmune diseases and increases the risk to develop multiple sclerosis. Here, we hypothesized that immune activation during IM triggers autoreactive immune responses. Antibody responses towards cellular antigens using a HEp-2 based indirect immunofluorescence assay and native myelin oligodendrocyte glycoprotein (MOG) using a flow cytometry-based assay were determined in 35 patients with IM and in 23 control subjects. We detected frequent immunoglobulin M (IgM) reactivity to vimentin, a major constituent of the intermediate filament family of proteins, in IM patients (27/35; 77%) but rarely in control subjects (2/23; 9%). IgG autoantibodies binding to HEp-2 cells were absent in both groups. In contrast, IgG responses to native MOG, present in up to 40% of children with inflammatory demyelinating diseases of the central nervous system (CNS), were detectable in 7/35 (20%) patients with IM but not in control subjects. Normalization of anti-vimentin IgM levels to increased total IgM concentrations during IM resulted in loss of significant differences for anti-vimentin IgM titers. Anti-MOG specific IgG responses were still detectable in a subset of three out of 35 patients with IM (9%), even after normalization to increased total IgG levels. Vimentin-specific IgM and MOG-specific IgG responses decreased following clinical resolution of acute IM symptoms. We conclude from our data that MOG-specific memory B cells are activated in subset of patients with IM.

## 1. Introduction

Epstein-Barr virus (EBV) is a gamma-herpesvirus that establishes a benign, lifelong infection in resting memory B cells in over 90% of the human population worldwide. In the industrialized world, about 50% of the population acquires EBV between one and five years of age, while another large percentage contracts the virus during adolescence [1]. Up to 77% of individuals, who acquire EBV in the second life decade or later, manifest symptomatic primary infection, known as infectious mononucleosis (IM) [2]. EBV infection, and in particular a history of IM, have been associated with the development of autoimmune diseases [3]. Several well-controlled epidemiological studies confirmed that a history of IM is associated with an approximately two-fold increased risk to develop multiple sclerosis (MS) later in life [3,4]. In addition, EBV seropositivity occurs more frequently in patients with systemic lupus erythematosus (SLE) compared to demographically matched healthy controls [5] and juvenile forms of these diseases appear to be particularly associated with EBV [6,7]. 

We hypothesized that strong immune activation during IM facilitates activation and expansion of autoreactive lymphocytes, thereby lowering the threshold for breakdown of self-tolerance to autoantigens. Therefore, we profiled autoreactive immunoglobulin M (IgM) and IgG responses to a panel of autoimmune disease-associated antigens in patients with IM.

## 2. Materials and Methods

Autoreactive antibody responses were determined in two independent cohorts of a total of 35 patients with IM and in 23 control subjects matched by age and gender (Table 1). Patients were recruited at the Department of Pediatrics, University Hospital Zurich (Zurich, Switzerland) (cohort 1) and Innsbruck (Austria) (cohort 2). Control subjects recruited in cohort 1 consisted of children with acute symptoms compatible with IM (fatigue, fever, sore throat, cervical lymphadenopathy) but seronegative for EBV-derived viral capsid antigen (VCA)-specific IgM and seropositive for EBV-derived nuclear antigen 1 (EBNA1)-specific IgG indicating acute upper respiratory tract but past EBV infection. Control subjects recruited in cohort 2 consisted of children with non-inflammatory diseases (migraine and other types of headaches not associated with inflammatory conditions and attention deficit hyperactivity disorder). The study was approved by the local Institutional Review Board (IRB StV29/06 University of Zurich, IRB AM4059 University of Innsbruck), and all subjects and/or their guardians provided informed consent. Blood samples were drawn at hospital admission, which was 3–28 days after onset of illness (Table 1). Samples derived from IM patients and controls in each cohort were obtained using the same procedures and stored under the same conditions. Heterophile antibodies of the IgM type directed against mammalian erythrocytes are associated with IM (Paul-Bunnell reaction), but their determination can result in false-negative and false-positive results. Here, IM was diagnosed based on the clinical presentation, presence of anti-VCA IgM antibodies and lack of IgG antibodies specific for EBNA1. Free viral DNA was detectable in all serum samples derived from IM patients but in none of the control samples. IgM and IgG responses to cellular antigens were determined using a Hep-2 based indirect immunofluorescence assay. IgM responses to vimentin were quantified by enzyme-linked immunosorbent assay (ELISA) according to the manufacturer’s recommendation (Vidia Ltd., Jesenice, Czech Republic). IgG responses to myelin oligodendrocyte glycoprotein (MOG) were determined using a fluorescence-activated cell sorting (FACS) live cell-based assay as described previously [9,10]. In brief, human oligodendroglial MO3.13 cells, which expresses phenotypic characteristics of primary oligodendrocytes, were transduced to express full-length human MOG [9]. Serum samples were incubated with MO3.13MOG+ cells which were subsequently stained with an Alexa Fluor 488-labeled goat anti-human IgG secondary antibody (Thermo Fisher Scientific, Reinach, Switzerland) [9,11]. Analysis of cell surface staining was determined by flow cytometry and MOG-specific IgG titers were defined by subtraction of mean of the fluorescence intensity (MFI) obtained with non-transduced MO3.13 control cells from the MFI obtained with transduced MO3.13 MOG+ cells (ΔMFI) [9,10]. The cutoff was set to 3 standard deviations above the mean ΔMFI of pediatric controls and samples were considered positive if they were above threshold at least 2 times out of 3 repeated experiments [9,11]. 

**Table 1:** Demographical characteristics of patients and control subjects.

	Cohort 1		Cohort 2	
	Controls	IM patients	Controls	IM patients
**Number**	10	22	13	13
**Gender (f/m)**	6/4	11/11	9/4	5/8
**Median Age (Range)**	9.5 (3-15)	12 (2-15)	16 (8-18)	16 (7-18)
**Duration of Symptoms**	NA	7-28 days	NA	3-21 days

## 3. Results

**Figure 1:**
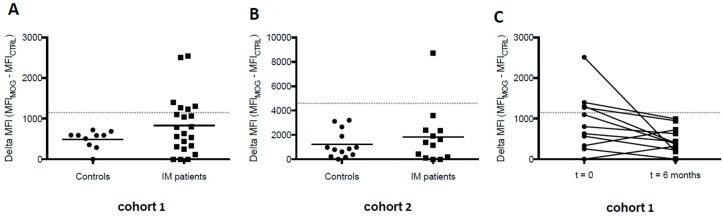
Elevated myelin oligodendrocyte glycoprotein (MOG)-specific immunoglobulin G (IgG) responses during acute infectious mononucleosis (IM). Serum anti-MOG IgG reactivity of control and IM patient sera from cohort 1 (**A**) and cohort 2 (**B**) was detected at time of diagnosis. The positivity threshold, designated with a grey dotted line, was determined by three standard deviations above the mean of the control samples; (**C**) MOG-specific IgG responses disappear six months post IM diagnosis.

MOG-specific IgG antibody responses were detected in a subgroup of IM patients (*n* = 7/35) but in none of the control subjects (*n* = 0/23) (Figure 1A,B). To evaluate whether MOG-specific IgG responses would remain elevated following clinical resolution of acute IM symptoms, we additionally determined antibody responses six months after onset of IM in cohort 1, in which samples from 11/22 patients were obtained longitudinally. MOG-specific IgG responses decreased in all patients and fell below cut off levels in those patients who exhibited positive MOG-specific responses during acute IM (Figure 1C). Thus, IgG antibodies specific for native MOG, indicative for central nervous system (CNS) demyelinating diseases in children, are transiently increased in a subgroup of patients with IM. None of the patients developed a clinical phenotype reminiscent of MOG-IgG-associated diseases such as acute disseminated encephalomyelitis (ADEM), MS, aquaporin-4-seronegative neuromyelitis optica spectrum disorder (NMOSD), isolated optic neuritis or transverse myelitis, or bilateral optic neuritis (BON) within the observation period of six months after onset of acute IM.

**Figure 2:**
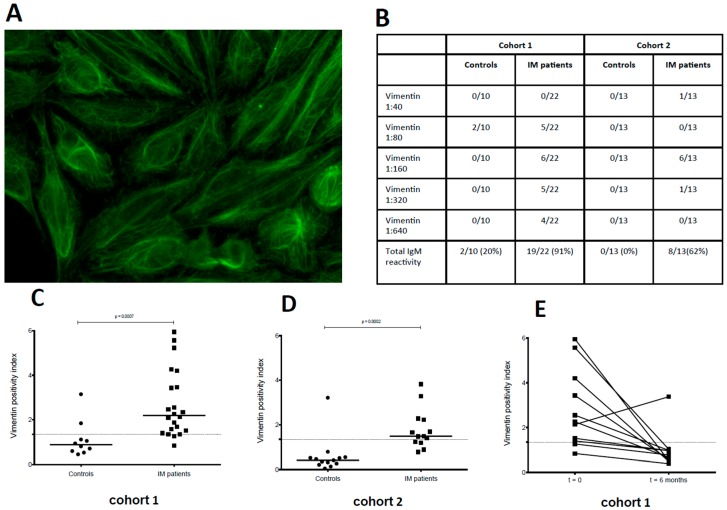
Transient IgM autoreactivity during acute IM. HEp-2 immunofluorescence staining was performed with serum derived from IM patients and control subjects from both cohorts. (**A**) HEp-2 positive vimentin-like staining of one representative IM patient; (**B**) Summary table of elevated IgM reactivity to vimentin during acute IM observed in both cohorts; (**C**) and (**D**) ELISA detection of anti-vimentin IgM autoantibodies in IM patients was significantly elevated compared to controls in both cohorts; (**E**) Longitudinal samples collected from IM patients in cohort 1 revealed decrease in anti-vimentin IgM antibodies six months post diagnosis of acute IM.

Using a Hep-2 based indirect immunofluorescence assay, we detected frequent IgM reactivity to reticular cytoplasmic antigens reminiscent of vimentin exclusively in IM patients (27/35; 77%) but rarely in control subjects (2/23; 9%) (Figure 2A,B). IgG autoantibodies binding to HEp-2 cells were absent in both groups. IgM reactivity towards vimentin could be confirmed by ELISA in both IM cohorts (Figure 2C,D). Longitudinal analysis of anti-vimentin IgM reactivity showed that these antibodies fell below the detection limit of the ELISA six months following acute IM in almost all patients studied (10/11; 91%). Thus, patients with acute IM showed elevated MOG-specific IgG and vimentin-specific IgM responses as compared with children with acute upper respiratory tract infections not associated with EBV infection (cohort 1) and with non-inflammatory disease conditions (cohort 2). Autoreactive antibody responses decreased following clinical resolution of acute IM symptoms.

**Figure 3:**
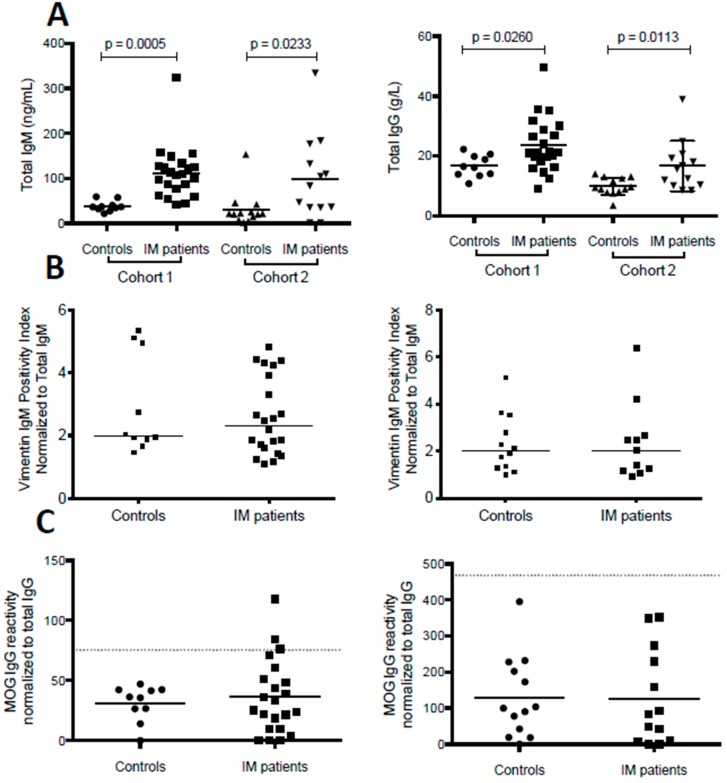
Vimentin-specific IgM and MOG-specific IgG autoantibody titers during acute IM following normalization to serum total IgM and serum total IgG. Total IgM and IgG (**A**) concentrations were measured in serum of IM patients and controls in both cohorts. Both IgM and IgG levels were significantly elevated during acute IM compared to controls; (**B**) Vimentin-specific IgM was normalized to total IgM concentrations, which led to loss of significant differences for vimentin-specific IgM titers between IM patients and controls in cohort 1 (left panel) and cohort 2 (right panel); (**C**) MOG-specific IgG responses were normalized to total IgG concentrations. Despite normalization to total IgG, three IM patients in cohort 1 presented with MOG IgG reactivity above the positivity threshold. The positivity threshold, designated with a grey dotted line, was set at three standard deviations above the mean of the respective control group.

During IM, activation of B lineage cells by EBV results in polyclonal immunoglobulin production and increased serum concentrations of immunoglobulin subclasses [12,13]. We found that IM patients from both cohorts showed higher total serum IgM and IgG levels as compared to controls (Figure 3A). Normalization of anti-vimentin IgM levels to total IgM concentrations resulted in a complete loss of significant differences for anti-vimentin IgM titers (Figure 3B). In contrast, anti-MOG specific IgG responses were still detectable in a subset of three out of 35 patients with IM (9%), even after normalization to total IgG levels (Figure 3C).

## 4. Discussion

IgM antibodies specific for autoantigens have previously been described to occur frequently during acute IM, to be highest soon after onset and to disappear during convalescence [14,15,16]. These antibodies were shown to bind to a number of antigens present in EBV-transformed B cells and to partly cross-react with the glycine-alanine repeat domain of EBNA1 [15]. Vimentin, a major constituent of the intermediate filament family of proteins, serves scaffolding functions within mesenchymal cells and has been implicated in facilitating cell transformation and latent EBV-derived membrane protein 1 (LMP1)-signaling during EBV infection [17,18] and IgM antibodies preferentially binding to vimentin and intermediate filaments have previously been detected in IM sera [19,20,21]. While we did not determine whether IgM antibodies to vimentin cross-react to EBV-encoded antigen, the loss of significance for vimentin reactivity after normalization to total IgM indicates that the occurrence of these antibodies reflect random polyclonal B cell activation during acute IM or represent low-affinity IgM antibodies which disappear during affinity maturation of virus-specific B cells [15,16]. In contrast, the detection of IgG responses to MOG during acute IM implies that the producing B lineage cells underwent affinity maturation and acquired memory function. IgG responses to MOG, localized on the outermost surface of myelin within the CNS, are found in up to 40% of children with CNS demyelinating diseases such as ADEM, MS, NMOSD, isolated optic neuritis or transverse myelitis, but not in control subjects and only rarely in adults with these disorders [8,9].

Latent EBV infection is thought to confer B cell survival advantages during antigen-driven selection by mimicking signals of T cell help and B cell receptor engagement through LMP1 and LMP2 expression [22]. However, EBV infection of memory B cells in patients with acute IM does not result in increased frequencies of cells specific for autoantigens including MOG [23] arguing against a role for EBV in assisting the survival of autoreactive B cells. Instead, memory B cells can differentiate into antibody-secreting plasma cells in response to polyclonal stimuli, such as Toll-like receptor 9 (TLR9) ligation [24,25,26]. EBV DNA, readily available during acute IM, stimulates TLR9 [27,28]. We infer from these data that IM-associated adjuvant effects such as TLR9 stimulation by EBV DNA might trigger polyclonal memory B cell stimulation and secretion of autoantigen-specific IgG. The occurrence of autoreactive IgG antibodies during symptomatic primary EBV infection could signal a propensity to develop autoimmune diseases in the future.
